# The Effects of Sprint vs. Resisted Sled-Based Training; an 8-Week in-Season Randomized Control Intervention in Elite Rugby League Players

**DOI:** 10.3390/ijerph18179241

**Published:** 2021-09-01

**Authors:** Jonathan Sinclair, Christopher James Edmundson, John Metcalfe, Lindsay Bottoms, Stephen Atkins, Ian Bentley

**Affiliations:** 1 Research Centre for Applied Sport, Physical Activity and Performance, Faculty of Allied Health and Wellbeing, School of Sport & Health Sciences, University of Central Lancashire, Preston PR1 2RA, UK; cjedmundson@uclan.ac.uk (C.J.E.); jmetcalfe1@uclan.ac.uk (J.M.); ibentley1@uclan.ac.uk (I.B.); 2 Centre for Research in Psychology and Sport Sciences, School of Life and Medical Science, University of Hertfordshire, Hatfield AL10 9AB, UK; l.bottoms@herts.ac.uk; 3 School of Health and Society, University of Salford, Manchester M5 4WT, UK; s.j.atkins@salford.ac.uk; 4 Wigan Warriors RLFC, Wigan WN5 0UH, UK

**Keywords:** rugby league, sprint, resisted sled training, agility, strength and conditioning

## Abstract

The aim of the current study was to examine the efficacy of resisted sled-based training compared to traditional unresisted sprint training in terms of mediating improvements in speed, agility, and power during an eight-week period of in-season training in elite rugby league players. Participants were randomly separated into either resisted sled or traditional sprint-based training groups and they completed an eight-week in-season training block with training prescribed based on the group to which they were assigned. Measures of 5 m, 10 m, and 20 m sprint times in addition to countermovement jump height and 505-agility test time were measured at baseline, four-weeks and eight-weeks. For sprint-based outcomes, although both groups improved significantly, there were no statistical differences between the two training methods. However, at the eight-week time point there were significant improvements in 505-agility test (sprint group: baseline = 2.45 and eight-weeks = 2.42 s/sled group: baseline = 2.43 and eight-weeks = 2.37 s) and countermovement jump (sprint group: baseline = 39.18 and eight-weeks = 39.49 cm/sled group: baseline = 40.43 and eight-weeks = 43.07 cm) performance in the sled training group. Therefore, the findings from this investigation may be important to strength and conditioning coaches working in an elite rugby league in that resisted sled training may represent a more effective method of sprint training prescription.

## 1. Introduction

Rugby league represents an intermittent collision team-based sport, characterized by bouts of high intensity running, physical collisions, and tackling, with intervening periods of reduced intensity activity [1]. A rugby league therefore relies on several components of athletic aptitude, including aerobic fitness, anaerobic fitness, muscular strength, power, speed, and agility in order to compete at elite level [2]. A rugby league requires players to be able to move quickly in order to position themselves effectively in both attack and defense [3], and previous analyses have importantly shown that speed is associated with increased tackling performance and has been shown to distinguish between playing levels [1,4,5]. Furthermore, increased lower body power has been shown to be associated with increased tackling ability [5] and is similarly able to differentiate between playing standards [6]. Similarly, in regards to agility, rugby league requires players to rapidly accelerate, decelerate, and change direction in both attack and defense, and previous investigations have confirmed that agility is able to differentiate between players of different ability [7]—highlighting clearly the importance of maximizing speed, agility, and power in an elite rugby league.

Strength and conditioning practitioners are able to develop the aforementioned parameters in a variety of different ways such as incorporating strength exercises, traditional sprint training, plyometric exercises, or with a more combined approach [8]. In recent years, resisted sprint training has received considerable attention in strength and conditioning literature and is now commonly adopted by practitioners as a means to improve speed and acceleration in running [9]. Resisted sprint training involves the athlete sprinting with an added load (i.e., resistance) using either a weighted sled, a weighted vest, or speed parachute. Resisted sprint training modalities are undertaken in a horizontal direction and involve the relevant muscles, velocities, and ranges of motion to those of uninhibited sprinting [9]. The most commonly adopted resisted sprint training approach is resisted sled training, whereby a sled is towed (using either a shoulder or waist harness) behind the athlete, and the external load is in the form of a weighted sled/the friction coefficient between sled and ground [10].

Resisted sled training has received considerable attention in a range of acute investigations within the field of strength and conditioning. Compared to un-inhibited sprinting (i.e., without towing a weighted sled); resisted sled towing has been shown to cause increases in trunk lean, contact time, knee flexion angle, propulsive impulse, peak braking forces; in addition to decreases in running velocity, mean vertical forces, peak hip flexion, swing phase duration, stride length, and stride frequency [11,12,13,14,15].

However, one of the most important factors in any resistance training exercise is the magnitude of the resistance itself, and sled loading can be determined using various strategies [9]. Sled loading strategies have varied greatly between investigations with loads as low as 5% and as high as 32.2% body mass having been examined within the literature. Lockie et al. [11] showed significant reductions in running velocity, stride length, and stride frequency between loadings of 12.6% and 32.2% body mass. Furthermore, Cronin et al. [13] confirmed that significant reductions in running velocity, stride length, and increases in contact time were evident between loadings of 15% and 20% body mass. In addition, Kawamori et al. [15] found significant reductions in running velocity in addition to increases in horizontal impulse and peak braking force between loadings of 10% and 30% body mass. Finally, Bentley et al. [8] showed that there were linear statistical decreases in running velocity and similar increases in knee flexion, contact time, and propulsive impulse between velocity reduction loadings of 10%, 15%, and 20%. Sled loading was initially considered relative to body mass; however, loadings based on body mass do not account for individual variations in strength, power, or technical ability [9]. Therefore, although more time-consuming, the most commonly adopted strategy is now to load the sled-based on reductions in sprint velocity, with Bentley et al. [8] advocating velocity reductions of 20%.

Furthermore, a number of randomized intervention analyses have considered the effects of resisted sled towing, on performance-based outcomes in a range of athletic disciplines. There has been a lack of consensus in the findings from intervention analyses concerning the effects of resisted sled training that are likely due to the population being examined, as well as the adopted loading strategy and the length of the intervention being tested [9]. Some investigations have shown that resisted sled training has no beneficial effect in relation to traditional unrestricted sprint training modalities. For example, Lockie et al. [16] examined the effects of a six-week resisted sled training intervention, loaded at 12.6% body mass, in comparison to resistance, plyometric and traditional sprint-based training groups. They showed that while resisted sled training produced significant improvements in 0–10 m sprint, reactive strength, and three repetition maximum squat performance, these improvements were not above and beyond those in the other training groups. In addition, McMorrow et al. [17] investigated the influence of six-weeks of heavy resisted sled training at 30% body mass compared to traditional sprint-based training in professional soccer players. The findings from this investigation showed that resisted sled training mediated improvements in both sprint and countermovement jump performance, but not above those shown in the traditional sprint training group. Finally, Clark et al. [18] studied the effects of a seven-week resisted sled training program using a 10% body mass loading strategy compared to weighted vests at 18.5% body mass and traditional sprint training groups in collegiate level lacrosse players. Their results revealed small improvements in sprint performance in the sprint training group, but only trivial improvements in the resisted sled and weighted vest groups, indicating that with regards to sprint performance, resisted sled training had no beneficial effect.

However, some investigations have shown that resisted sled-based training is effective in mediating statistical improvements in performance outcomes. Spinks et al. [19] examined an eight-week resisted sled-based training program with 10% velocity reduction, in comparison to sprint training and control groups in soccer, rugby union, and Australian football players. Their findings showed that although resisted sled training significantly improved 0–15 m sprint and countermovement jump performance this training modality was not more effective than sprint training. However, resisted sled training did mediate significant improvements in reactive strength ability during the drop jump above any beyond those in the sprint and control groups. Harrison and Bourke [20] examined the effects of a six-week resisted sled training intervention at 13% body mass compared to a control group. Their findings confirmed that compared to controls, the resisted sled training group had significant improvements in time to 5 m and countermovement jump height after the six-week intervention. Similarly, West et al. [21] investigated the influence of a six-week resisted sled-based intervention at 12.6% body mass compared to a traditional unresisted sprint training group. Their observations showed that although both groups mediated statistical improvements in sprint performance, adaptations in the sled training group were significantly greater. Lahti et al. [22] investigated whether two heavy resisted sled training conditions, i.e., 50% and 60% velocity reduction, affected sprint performance, kinetics, sagittal plane kinematics, and spatiotemporal parameters over nine-weeks compared to traditional training in professional male soccer players. This investigation showed that both sled-based training groups mediated significant improvements in 5, 10, 20, and 30 m sprint performance in addition to indices of mechanical efficiency, peak power, and peak force that were not present in the traditional training group. Morin et al. [23] examined 30 m sprint performance and kinetic outputs one week before (baseline) and one (post-test), two, three, and four weeks following a 10-week resisted sprint-based intervention. In this investigation the prescribed load was undertaken based on the apex of their velocity-power relationship, which corresponded to 90% ± 10% body mass and statistical comparisons were undertaken based on both baseline vs. post-test and baseline vs. the testing week associated with peak performance. The findings showed only trivial-small improvements from baseline to post-test but revealed much larger small-moderate improvements from baseline to peak performance, which importantly was not shown to be the post-test time point for any of the outcome measurements.

At the current time, there is no research concerning the efficacy of resisted sled-based training compared to traditional unresisted sprint approaches in elite rugby league players. Meaning that a randomized control investigation in this population concerning the effects of resisted sled training would be of clear practical relevance to strength and conditioning coaches and practitioners working within rugby league. Therefore, the aim of the current study was to examine using a randomized trial; the efficacy of resisted sled-based training compared to traditional unresisted sprint training in terms of mediating improvements in speed, agility, and power during an eight-week period of in-season training in elite rugby league players. A study of this nature may inform both strength and conditioning coaches and rugby league athletes themselves, regarding the most effective approach for the prescription of sprint-based training.

## 2. Materials and Methods

### 2.1. Participants

Twenty-eight male professional rugby league players (mean ± standard deviation: age: 18.8 ± 0.6 years: body mass: 87.6 ± 11.4 kg: stature: 182.2 ± 5.5 cm and BMI: 26.3 ± 2.6 kg/m^2^) contracted to a super-league club in the United Kingdom, volunteered to take part in this experiment. Two players had to withdraw from the investigation either due to injury of illness. All participants were professional players from a Super League squad and had at least 3 years of rugby league-based sprint training experience. Participants provided written informed consent and ethical approval (REF: BuSH 202) was obtained from the University of Central Lancashire, in accordance with the principles documented in the Declaration of Helsinki.

### 2.2. Procedure

Participants were allocated to either the sled or sprint-based group using a computer program (Random Allocation Software). Both training intervention groups were incorporated into the players’ traditional in-season program. For consistency all participants were exposed to the same standardized training program in the 4-weeks immediately prior to this study. The interventions were scheduled over an 8-week period, during this window the participants’ normal training program continued (involving 3 × 45 min gym and 3 × 70 min technical sessions per week—Table 1).

The training programs were broken up into 2× repeated 4-week blocks and were identical other than the sled or sprint protocols. Once the first four-week training block was complete, this was repeated with adjusted exercise loadings. The sled and sprint-based training protocols were undertaken twice per week throughout the 8-week intervention on Tuesdays and Thursdays within the scheduled gym sessions (Table 1 and Table 2). All sprint-based training sessions began with a standardized warm-up consisting of jogging (5 min), dynamic stretching (5 min), and several short sprints building up to maximum intensity (4 × submaximal and 2 × maximal). The intervention sprints were completed in an indoor sports hall with a hard rubber surface (μ = 0.38), which was established using the protocol of Linthorne and Cooper, [24].

### 2.3. Sled Towing Group

Participants completed 3 × 20 m sled tows with 2 min recovery between each sprint. After the third sprint participants had 3 min recovery before repeating the procedure twice more. These sets and reps were similar to those of previous analyses [20,21]. Compared to the other investigations which used a much higher volume of running, these strategies minimize fatigue and therefore suit the nature of a concurrent program [25]. Sleds were loaded to produce reduce velocity by 20%, as recommended by our previous work [8]. Sled loadings were determined in order to produce the required 20% velocity reduction over 10 m and calculated during a familiarization session one-week prior to the baseline testing session and recalculated halfway through the intervention. Sled loadings corresponding to 25.0% ± 3.4% and 26.9% ± 4.6% body mass in weeks 1–4 and 5–8, respectively.

### 2.4. Sprint Training Group

Participants completed 3 × 20 m un-inhibited sprints with 2 min recovery between each sprint. After the third sprint, participants had 3 min recovery before repeating the procedure twice.

### 2.5. Testing Procedures

All participants were tested at baseline, 4-weeks and 8-week time points and identical protocols were followed before each testing session. All tests were carried out within a single testing session in a randomized order, participants were given 2 min recovery within tests and 4 min between different tests. All testing was conducted on a Monday and commenced following a period of 24 h rest as players do not train on Sunday (Table 1). Participants were instructed not to consume any alcohol during this period and continue with their typical training day diet. All participants completed a familiarization session during which all testing protocols were practiced until participants were confident.

### 2.6. 5, 10 and 20 m Sprints

**Figure 1:**
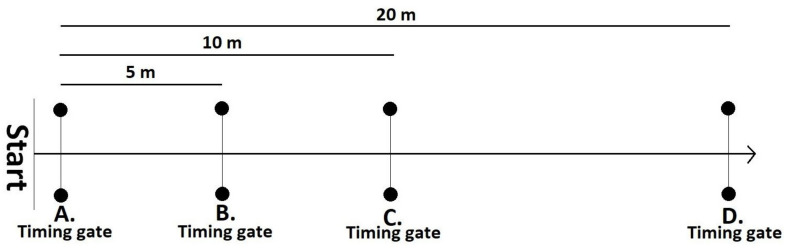
Diagram of 5, 10, and 20 m sprint test protocol.

The testing session began with a standardized warm-up consisting of jogging (5 min), dynamic stretching (5 min), and a number of short sprints building up to maximum intensity (4 × submaximal and 2 × maximal). On completion of the warm-up participants completed 3 × 20 m sprints from a standing staggered stance with their non-dominant foot forward through the electronic timing gate system (Fusion Sports, SmartSpeed, Australia). Participants started 0.3 m behind the starting point and timing gates were positioned at 0 (point A), 5 (point B), 10 (point C), and 20 m (point D) (Figure 1). Participants were instructed to start when they were ready and to sprint through the 5 m past the final gate. The fastest time to each of the three distances out of all of the attempts was extracted for data analysis.

### 2.7. Counter Movement Jump

The counter movement jump (CMJ) began with participants standing tall with hands on their hips. They were instructed to perform a countermovement by simultaneously flexing the hips and knees to a self-selected depth then explosively jumping as high as possible, with the hands remaining on the hips throughout. Participants were instructed to land in the same position on the mat with a toe first contact. The jumps were performed on the electronic jump mat (Fusion Sports, SmartSpeed, Brisbane, Australia) which utilized flight time to calculate jump height. All participants performed 3 jumps with 3 min rest in between and the largest jump was recorded and utilized in for data analysis.

### 2.8. 505-Agility Test

**Figure 2:**
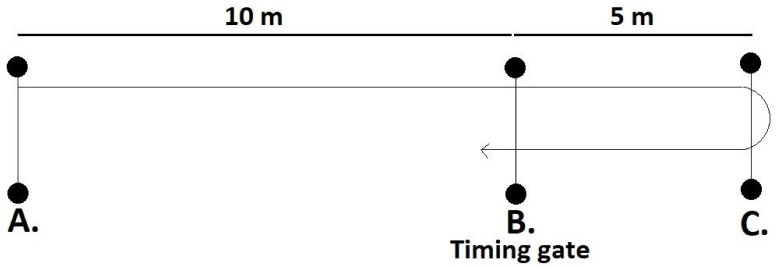
Diagram of 505-agility test protocol.

Participants were assessed using a single timing gate (Fusion Sports, SmartSpeed, Brisbane, Australia). During the 505-agility test (Figure 2) the participants started 10 m from the timing gate (15 m from the turning line—point A) and they sprinted through the timing gate (point B) before turning on the following line (point C) and accelerating back through the timing gate. Participants were instructed to place one foot over the line as they performed the 180-degree turn. The time was recorded from when participants first ran through the timing gate and stopped when they returned through the same timing gate. Each participant performed 2 trials turning on each leg (4 total) and the fastest trial for each leg was used during data analysis.

### 2.9. Analyses

Comparisons between participant characteristics at baseline between the two groups were undertaken using linear mixed models, with group modelled as a fixed factor and random intercepts by participants [26]. In order to examine whether there was an effect of time, i.e., whether there were differences between the three experimental time points across both groups, mediated by the 8-week intervention, repeated measures linear mixed models were used with time (i.e., Baseline, 4-weeks and 8-weeks) modelled as a fixed factor and random intercepts by participants [26]. Furthermore, in order to determine differences between the two training groups at the 4- and 8-week time points, linear mixed models with group modelled as a fixed factor and random intercepts by participants were adopted adjusted for baseline values modelled as continuous fixed covariates [27]. For linear mixed models the mean difference (b), t-value and 95% confidence intervals of the difference are presented and statistical significance for all analyses was accepted as the *p* < 0.05 level. All analyses were conducted using SPSS v27 (IBM, SPSS, New York City, USA).

## 3. Results

### 3.1. Baseline Characteristics

There were no significant differences between groups at baseline for age (b = 0.28, (95% CI = −0.18–0.75), t = 1.27, *p* > 0.05), body mass (b = 3.75, (95% CI = −5.55–13.04), t = 0.83, *p* > 0.05), stature (b = 0.64, (95% CI = −3.89–5.16), t = 0.29, *p* > 0.05), or BMI (b = 0.94, (95% CI = −1.14–3.02), t = 0.91, *p* > 0.05) (Table 3).

### 3.2. 5 m Sprint

There was a main effect of time showing a significant improvement in 5 m sprint performance between baseline and four-weeks (b = 0.03, (95% CI = 0.009–0.05), t = 3.08, *p* < 0.05) and between baseline and eight-weeks (b = 0.05, (95% CI = 0.025–0.07), t = 4.16, *p* < 0.05). There were no differences in performance between four- and eight-weeks (b = 0.02, (95% CI = −0.003–0.04), t = 1.73, *p* > 0.05). However, there were no significant differences between the sprint and sled training groups at either four-weeks (b = 0.01, (95% CI = −0.04–0.02), t = 0.60, *p* > 0.05) or eight-weeks (b = 0.03, (95% CI = −0.08–0.02), t = 1.17, *p* > 0.05) (Table 4).

### 3.3. 10 m Sprint

There was a main effect of time showing a significant improvement in 10 m sprint performance between baseline and four-weeks (b = 0.03, (95% CI = 0.004–0.05), t = 2.43, *p* < 0.05) and between baseline and eight-weeks (b = 0.04, (95% CI = 0.02–0.07), t = 3.42, *p* < 0.05), although there were no differences in performance between four- and eight-weeks (b = 0.02, (95% CI = −0.008–0.04), t = 1.41, *p* > 0.05). There were no significant differences between the sprint and sled training groups at either four-weeks (b = 0.007, (95% CI = −0.05–0.04), t = 0.31, *p* > 0.05) or eight-weeks (b = 0.04, (95% CI = −0.08–0.009), t = 1.67, *p* > 0.05) (Table 4).

### 3.4. 20 m Sprint

There was a main effect of time showing a significant improvement in 20 m sprint performance between baseline and four-weeks (b = 0.03, (95% CI = 0.001–0.05), t = 2.19, *p* < 0.05) and between baseline and eight-weeks (b = 0.03, (95% CI = 0.03–0.08), t = 4.03, *p* < 0.05), although there were no differences in performance between four- and eight-weeks (b = 0.03, (95% CI = −0.05–0.001), t = 1.97, *p* > 0.05). There were no significant differences between the sprint and sled training groups at either four-weeks (b = 0.02, (95% CI = −0.07–0.03), t = 0.95, *p* > 0.05) or eight-weeks (b = 0.04, (95% CI = −0.10–0.02), t = 1.37, *p* > 0.05) (Table 4).

### 3.5. Counter Movement Jump

There was a main effect of time showing a significant improvement in CMJ performance between baseline and four-weeks (b = 0.88, (95% CI = 0.07–1.69), t = 2.18, *p* < 0.05) and between baseline and eight-weeks (b = 1.47, (95% CI = 0.66–2.28), t = 3.66, *p* < 0.05), although there were no differences in performance between four- and eight-weeks (b = 0.59, (95% CI = −0.21–1.40), t = 1.48, *p* > 0.05). There were no significant differences between the sprint and sled training groups at four-weeks (b = 1.41, (95% CI = −0.42–3.23), t = 1.59, *p* > 0.05) but at eight-weeks CMJ height was significantly greater in the sled training group (b = 2.32, (95% CI = 0.85–3.79), t = 3.24, *p* < 0.05) (Table 4).

### 3.6. 505-Agility Test

There was a main effect of time showing a significant improvement in 505-agility test performance between baseline and eight-weeks (b = 0.05, (95% CI = 0.02–0.08), t = 3.52, *p* < 0.05), although there were no differences in performance between baseline and four-weeks (b = 0.02, (95% CI = −0.004–0.05), t = 1.74, *p* > 0.05) or between four- and eight-weeks (b = 0.02, (95% CI = −0.05–0.003), t = 1.78, *p* > 0.05). There were no significant differences between the sprint and sled training groups at four-weeks (b = 0.03, (95% CI = −0.06–0.007), t = 1.61, *p* > 0.05) but at eight-weeks 505-agility test performance was significantly greater in the sled training group (b = 0.04, (95% CI = 0.002–0.08), t = 3.15, *p* < 0.05) (Table 4).

## 4. Discussion

The current investigation aimed to examine the efficacy of resisted sled-based training compared to traditional unresisted sprint training in terms of mediating improvements in speed, agility and power during an eight-week period of in-season training in elite rugby league players. This represents the first investigation in this population to examine the potential benefits of resisted sled-based training using a randomized trial and may thus provide important information to strength and conditioning coaches working in elite rugby league regarding the most effective approach for the prescription of sprint-based training.

In relation to the observations from the sprint-based testing, the current investigation showed firstly across both groups that significant improvements in all three sprint distances were mediated between baseline and both the four- and eight-week intervention time points. This observation was to be expected in that significant improvements in sprint performance were noted as a result of a sprint intervention either in the form of unresisted or indeed resisted training interventions [21]. However, in relation to the sprint-based outcomes, it was importantly revealed that there were no statistical differences between the two sprint training methods. This observation concurs with those of Lockie et al. [16], McMorrow et al. [17], and Spinks et al. [19]. However, it differs from those of West et al. [21] and Lahti et al. [22] who found significant improvements in their resisted sled groups using intervention durations of six- and nine-weeks and loading stimuli of 12.6% body mass and 50–60% velocity reduction, respectively. It is conceivable, as proposed by Morin et al. [23], that the post intervention measurement time, i.e., eight-weeks, utilized in the current investigation did not correspond to the players’ respective time of peak performance after the training overload, and thus it is possible that peak sprint performance occurred at a different instant than the experimental post-measurement time point. It is clear that future investigations should seek to adopt a repeated follow-up study design to better understand the training adaptations mediated by resisted sled training. Regardless, this observation indicates that in terms of group-based outcomes neither approach examined in this study appeared to be superior in terms of mediating improvements in sprint performance, immediately after the eight-week intervention.

Similar to the sprint-based measures, the findings in relation to the countermovement jump and 505-agility tests showed that there were significant improvements across both groups detected as a function of the eight-week intervention. Importantly, however, it was also revealed that the resisted sled training group was successful in mediating significant improvements in these parameters, above and beyond those shown in the unresisted sprint group. This observation opposes those of Alcaraz et al. [14], McMorrow et al. [17] and Spinks et al. [19] who found no between group differences in jump or agility-based outcomes measures. However, this finding agrees with those shown by Harrison and Bourke [20] who showed improvements in jump performance in their resisted sled group, but there has yet to be an investigation showing improvements in agility attributable to resisted sled-based interventions. It has been proposed that resisted sled training enhances eccentric strength of the leg extensor muscles during the deceleration phase of ground contact and also increases muscle and leg stiffness [21], potentially mediating reductions in ground-contact times and greater utilization of the stretch shorten cycle [28]. As enhanced limb stiffness has been shown to be linearly associated with both countermovement jump and agility-based parameters [29,30], which may explain the increases in agility and countermovement jump performance in the resisted sled training group. Regardless, as agility and lower body power have been shown to be important performance-based outcomes in rugby league [6,7], this indicates that the resisted sled training group may represent a more effective method of sprint training prescription in elite rugby players.

A potential drawback to the current investigation is that only resisted sled and sprint training groups were examined. Future examination of a third intervention group, exploring the combined effects of resisted sled and sprint training, may better inform the programming of team-sport athletes. Furthermore, the results of this study are likely specific to the highly trained population that was examined and thus may not be applicable to recreational athletes. Finally, the eight-week intervention period utilized in the current study may not have been sufficient for all training adaptations to manifest as strength, hypertrophy, and neural-based adaptations to training are mediated at different rates [31]. Therefore, future investigations may wish to explore longer intervention periods, although this may be problematic in elite rugby league due to the challenging nature of the playing season.

## 5. Conclusions

The current study adds to the current literature in strength and conditioning by examining the efficacy of resisted sled-based training compared to traditional unresisted sprint training during an eight-week period of in-season training in elite rugby league players. The current investigation showed that whilst there were no differences between the two groups in terms of improvements in sprint performance, the resisted sled training group was associated with significant improvements in both agility and countermovement jump performance. These observations are of clear practical relevance to strength and conditioning coaches and practitioners. Agility and explosive power are known to be important to overall performance in elite rugby league. Therefore, findings from the current investigation suggest that resisted sled training may represent a more effective method of sprint training prescription to be implemented by strength and conditioning coaches in elite rugby league.

## Figures and Tables

**Table 1:** Weekly training details for all participants.

	Monday	Tuesday	Wednesday	Thursday	Friday	Saturday	Sunday
**am**	Gym	Gym	Off	Gym		Match	Off
**pm**	Match review session	Technical session		Technical session	Technical session		

**Table 2:** Training program information for the ‘Gym’ sessions outlined in Table 1.

	Monday (Full Body Strength)			
	Week 1	Week 2	Week 3	Week 4
**Exercise**	**Sets × repetitions**			
Pistol squats	3 × 6	3 × 6	3 × 6	3 × 6
SB curls	3 × 6	3 × 6	3 × 6	3 × 6
Military press	4 × 8	4 × 8	4 × 8	4 × 8
Weighted pull up	4 × 8	4 × 8	4 × 8	4 × 8
Banded walks	3 × 5 m	3 × 5 m	3 × 5 m	3 × 5 m
Bench bridges	3 × 8	3 × 8	3 × 8	3 × 8
Roll outs	4 × 6	4 × 6	4 × 6	4 × 6
YTV	4 × 4	4 × 4	4 × 4	4 × 4
	**Tuesday (full body strength)**			
	**Week 1**	**Week 2**	**Week 3**	**Week 4**
**Exercise**	**Sets × repetitions**			
***Sprints (sled or sprint group)***	**9 × 20 m**	**9 × 20 m**	**9 × 20 m**	**9 × 20 m**
Squats	4 × 8	4 × 8	4 × 8	4 × 8
Lateral lunges	4 × 4	4 × 4	4 × 4	4 × 4
Dumbbell bench	4 × 8	4 × 8	4 × 8	4 × 8
Bent over row	4 × 8	4 × 8	4 × 8	4 × 8
Reverse fly’s	4 × 10	4 × 10	4 × 10	4 × 10
Box step up’s	3 × 6	3 × 6	3 × 6	3 × 6
RDLS	3 × 6	3 × 6	3 × 6	3 × 6
	**Thursday (full body power)**			
	**Week 1**	**Week 2**	**Week 3**	**Week 4**
**Exercise**	**Sets × repetitions**			
***Sprints (sled or sprint group)***	**9 × 20 m**	**9 × 20 m**	**9 × 20 m**	**9 × 20 m**
Drop snatch	4 × 5	4 × 5	4 × 5	4 × 5
Squat jumps	4 × 5	4 × 5	4 × 5	4 × 5
SL box drives	4 × 6	4 × 6	4 × 6	4 × 6
Medicine ball slams	5 × 6	5 × 6	5 × 6	5 × 6
Bench throws	5 × 6	5 × 6	5 × 6	5 × 6
Hanging leg raises	5 × 4	5 × 4	5 × 4	5 × 4

**Table 3:** Participant baseline characteristics (mean ± SD) from each group.

	Sprint	Sled
N (completed)	13	13
Age (y)	18.7 ± 0.6	18.9 ± 0.5
Stature (cm)	182.5 ± 6.1	181.8 ± 5.1
Body mass (kg)	89.5 ± 11.4	85.7 ± 11.5
BMI (kg/m^2^)	26.8 ± 2.4	25.9 ± 2.7

**Table 4:** Outcomes (Mean ± SD) from as a function of each training group.

	Sprint						Sled						Difference from Baseline		Difference between Groups	
	Baseline		4-Weeks		8-Weeks		Baseline		4-Weeks		8-Weeks		4-Weeks	8-Weeks	4-Weeks	8-Weeks
	**Mean**	*SD*	**Mean**	*SD*	**Mean**	*SD*	**Mean**	*SD*	**Mean**	*SD*	**Mean**	*SD*				
5 m sprint (s)	1.02	0.06	1.00	0.07	0.99	0.06	1.03	0.07	1.00	0.05	0.97	0.08	*	*		
10 m sprint (s)	1.76	0.08	1.74	0.08	1.74	0.07	1.77	0.06	1.74	0.08	1.70	0.06	*	*		
20 m sprint (s)	3.03	0.12	3.01	0.11	2.99	0.11	3.01	0.10	2.97	0.10	2.94	0.11	*	*		
CMJ (cm)	39.18	6.59	39.34	6.70	39.49	6.75	40.43	3.87	42.02	5.18	43.07	4.55	*	*		*
505 (s)	2.45	0.07	2.44	0.07	2.42	0.06	2.43	0.11	2.40	0.08	2.37	0.06		*		*

* = statistical significance.
